# Exploring preferences and decision-making about long-acting injectable HIV pre-exposure prophylaxis (PrEP) among young sexual minority men 17–24 years old

**DOI:** 10.1038/s41598-023-32014-8

**Published:** 2023-03-29

**Authors:** Steven A. John, Juan P. Zapata, Madeline Dang, Benedikt Pleuhs, Andrew O’Neil, Sabina Hirshfield, Jennifer L. Walsh, Andrew E. Petroll, Katherine G. Quinn

**Affiliations:** 1 Health Intervention Sciences Group/Center for AIDS Intervention Research, Department of Psychiatry and Behavioral Medicine, Medical College of Wisconsin, 2071 N. Summit Avenue, Milwaukee, WI 53202 USA; 2 Department of Psychology, Marquette University, Milwaukee, WI USA; 3 Department of Psychiatry and Behavioral Sciences, University of Washington, Seattle, WA USA; 4 Department of Medicine, STAR Program, SUNY Downstate Health Sciences University, New York, NY USA

**Keywords:** Disease prevention, Public health

## Abstract

Intramuscular cabotegravir for long-acting injectable HIV pre-exposure prophylaxis (i.e., LAI-PrEP) was approved by the U.S. FDA in 2021. We sought to explore LAI-PrEP decision-making among a nationwide sample of young sexual minority men (YSMM) 17–24 years old. In 2020, HIV-negative/unknown YSMM (n = 41) who met CDC criteria for PrEP were recruited online to participate in synchronous online focus groups eliciting preferences and opinions about LAI-PrEP, as well as the impact of a potential self-administered option. Data were analyzed using inductive and deductive thematic analysis with constant comparison. Preferences and decision-making about LAI-PrEP varied widely among YSMM, with participants frequently comparing LAI-PrEP to oral PrEP regimens. We identified five key themes related to LAI-PrEP decision-making including concerns about adherence to PrEP dosing and clinic appointments, awareness and knowledge of PrEP safety and efficacy data, comfort with needles, minimizing PrEP stigma, and considerations of self-administration. YSMM acknowledged more PrEP options as beneficial to supporting uptake and persistence.

## Introduction

HIV incidence is declining in the U.S. largely due to advancements in biomedical HIV treatment and prevention^1^. Public health researchers found antiretroviral therapy to provide protection against HIV before exposure, which is referred to as HIV pre-exposure prophylaxis (PrEP). Introduced and approved by the U.S. Food and Drug Administration (FDA) in 2012^2^, HIV pre-exposure prophylaxis (PrEP) is highly effective in preventing HIV using oral dosing strategies, including once-daily^3–8^ and event-driven (i.e., “2–1–1”) dosing using tenofovir disoproxil fumarate and emtricitabine^9,10^, which is now available as generic. An additional drug formulation was also FDA approved in 2019 (i.e., tenofovir alafenamide and emtricitabine), but rigorous data are not yet available for event-driven dosing or for vaginal/frontal sex exposure. Aligned with advancements in antiretroviral therapy for HIV treatment^11^, phase 3 clinical trials of intramuscular cabotegravir were initiated in 2016^12,13^. These studies evaluated the use of cabotegravir administered by intramuscular injection in the gluteus muscle every 8 weeks. Intramuscular cabotegravir was found to provide comparable protection against HIV compared to daily oral PrEP within non-inferiority trials^12,13^. In fact, cisgender men who have sex with men and transgender women randomized to receive intramuscular cabotegravir had a 66% reduction in HIV incidence compared to those who received daily oral PrEP^13^, suggesting intramuscular cabotegravir may offer better protection than daily oral PrEP, which is well-established as highly effective by multiple clinical trials and demonstration projects^14–19^.

The introduction of intramuscular cabotegravir has the *potential* to increase PrEP uptake and persistence based on research with several priority populations for HIV prevention. First, intramuscular cabotegravir may be more acceptable for some individuals^20–30^. The use of oral dosing strategies has been identified as a barrier for some individuals when considering whether to initiate and their ability to adhere to oral PrEP^31,32^. More options provide greater patient choice and contextual fit analogous to contraception, where individuals have options for birth control that include daily oral pills, long-acting injectables, patches, subdermal implants, and intrauterine devices, among others^33^. Second, intramuscular cabotegravir only requires a single injection every 8 weeks, which could result in fewer concerns about adherence and subsequent effectiveness. Daily pill adherence has been identified as a barrier to PrEP uptake and persistence^31,32^, and providers have likely withheld prescription of oral PrEP from priority patients because of concerns about adherence^34^. Third, the use of intramuscular cabotegravir could be more discreet option, since dosing only occurs 6 times per year as opposed to daily with oral PrEP. This could potentially provide more confidentiality and reduce stigma among patients. HIV and PrEP stigmas are pervasive and a large barrier to PrEP uptake and persistence^35^, with individuals worrying about being considered HIV-positive or erroneously stereotyped as promiscuous for using PrEP^36^. As such, intramuscular cabotegravir offers another PrEP dosing mechanism to support HIV prevention, but research is limited on patient decision-making about LAI-PrEP.

The Centers for Disease Control and Prevention endorsed intramuscular cabotegravir for PrEP in the 2021 update of their PrEP guidelines^37^, nearly concurrent with the FDA’s approval of intramuscular cabotegravir^38^, yet there is a growing call for research to support patient access and decision-making to improve implementation^39^. Young gay, bisexual, queer, and other men who have sex with men—collectively referred to herein as young sexual minority men (YSMM)—are a priority population for HIV prevention based on startling HIV disparities compared to their heterosexual peers in the U.S.^1^. Although roll-out of intramuscular cabotegravir should be agnostic based on patient demographics to avoid further stigmatizing of PrEP use^40^, preferences and decision-making may vary among priority populations. In preparation for implementation of intramuscular cabotegravir as long-acting injectable PrEP (i.e., LAI-PrEP), we sought to explore LAI-PrEP decision-making among a nationwide sample of YSMM 17–24 years old.

## Methods

### Participants and procedures

As described previously^41,42^, participants were recruited online from social media and men-for-men geosocial networking apps between March and September 2020 to participate in online synchronous focus group discussions centered around topics of HIV testing and prevention. Ads were designed to target and oversample Black and Latino YSMM. All ads featured images of two men, including a variety of young adult couples of various races and ethnicities. Fraudulent responses to the screening survey were minimized by excluding any information on eligibility criteria from study advertisements and referral mechanisms, using the “prevent ballot box stuffing” feature in Qualtrics to prevent multiple responses, offering no incentive for completion of the brief (~ 5–10 min) screening survey and its associated online consent procedure, and using a delayed invitation procedure for the online focus group discussions to avoid attempts at determining the study’s eligibility criteria^43^. To further ensure data integrity, duplicates were checked using a procedure of comparing contact information (i.e., name, email, phone number) and IP addresses.

To be eligible to participate in an online focus group discussion, participants were required to: (1) be 17–24 years old; (2) identify as male (inclusive of transgender men); (3) report one or more male sexual partners in the past 6 months, including those who identified as transgender; (4) self-report HIV-negative or unknown status; (5) report sexual behavior meeting Centers for Disease Control and Prevention guideline criteria for PrEP^44^, which included past 6 month behavior of condomless anal sex (CAS) with a casual male partner, CAS with a main partner living with HIV or of unknown status, CAS with an HIV-negative main partner who reports CAS with other male partners, and/or having a recent history of bacterial sexually transmitted infection; and (6) reside in the U.S. Individuals who screened eligible received an email invitation to participate. Eligible participants who replied to our email invitation were then asked to complete an online consent procedure. Agreement-to-participate was obtained through Qualtrics using a guided procedure that described the study’s purpose, procedures, and other critical components. Participants were encouraged to email or call to get clarification on any questions prior to continuing, and several participants emailed with questions about privacy protections. Participants then completed a brief quiz to ensure adequate comprehension of the critical components of consent, including the voluntary nature of the study, risks and benefits to participation, and confidentiality of all data collected. Participants then agreed-to-participate online, and a copy of the study’s informational letter was emailed to the address of their choosing. A waiver of guardian permission was obtained for those considered minors. Participants were then scheduled for online group discussion(s), with 6–12 individuals invited per group.

Nine synchronous online focus groups were administered by a team of 2–3 researchers via online chat. Specifically, we used the real-time web-based meeting platform Adobe Connect for the online group discussions, which allowed between-participant anonymity with pre-selected usernames and no video recording. Online focus group discussions are a valid method of qualitative data collection for sensitive topics, which maintain fidelity of themes identified compared to in-person focus group discussions^45,46^. Online focus groups were about 90 min in duration, and participants were compensated with a $40 e-gift card. All text data were saved for analysis.

### Focus group content

A semi-structured focus group guide was developed to elicit preferences, opinions, barriers, facilitators, and decision-making about a variety of topics related to HIV testing and prevention, including HIV testing and self-testing, PrEP, post-exposure prophylaxis, eHealth intervention characteristics, and online focus group discussion experiences. Data from these focus groups were previously used to report barriers and facilitators to HIV prevention in the context of the COVID-19 pandemic^42^. Data used for this analysis centered around LAI-PrEP decision-making. More specifically, participants were provided with the following prompt to initiate the discussion: *“Researchers are currently working on a new form of PrEP that could be delivered by injection. If someone only had to get a shot once every 8 or more weeks to receive PrEP, do you think people would prefer that method of PrEP over a pill? Why or why not? What type of concerns do you think people will have about receiving PrEP by injection?”* With the goal of eliciting opinions about future implementation models of LAI-PrEP, participants were also asked about a self-injection option with the following prompt: *“If you could inject yourself rather than going to a provider every 8 weeks, would that increase your willingness to use it?”.*

### Data analysis

Descriptive statistics were used to characterize the sample using screening survey data. Transcripts were coded using MAXQDA by the first and second authors trained in qualitative methods using a three-stage analytic coding strategy including open, axial, and selective coding^47^. First, a list of a priori codes was developed in advance by the first and second author on topics addressed by the focus group guide. Codes were created by noting overlapping concepts in the transcripts and developing code definitions that represented the data. Each transcript was coded and reviewed separately to ensure adequate application of codes. During the initial analytic phase, each analyst separately coded the same randomized transcript with the codebook and inconsistencies were discussed until agreement was reached. Coded focus groups were then analyzed using thematic content analysis^48^ to highlight patterns and identify meaning of the data.

### Ethics approval and Informed consent

All procedures performed in studies involving human participants were in accordance with the ethical standards of the institutional and/or national research committee and with the 1964 Helsinki declaration and its later amendments or comparable ethical standards. All study procedures were approved by the Institutional Review Board of the Medical College of Wisconsin. This study met the Medical College of Wisconsin Institutional Review Board’s definition of “minimal risk” and a waiver of informed consent was granted. All participants agreed to participate after completion of a guided procedure using Qualtrics that described the study’s purpose, procedures, and other critical components, as well as a capacity-to-consent procedure. A waiver of guardian permission was obtained for those considered minors; states have varying ages for which minors are legally defined. Agreement-to-participate procedures were the same regardless of age given the waiver of guardian permission.

## Results

Online recruitment in 2020 yielded 133 unique YSMM eligible to participate in our online focus group discussions. Of the 133 YSMM who screened eligible, 118 provided contact information and were invited to enroll. Fifty-five YSMM agreed to participate, and 41 ultimately participated in an online focus group discussion. A total of nine focus group discussions were conducted spanning April-September 2020, with 3–7 participants in each group. Participants were predominately (85%) cisgender men, with six transgender men participating in focus group discussions. The sample was majority non-Hispanic Black (27%) or Latino (29%), with 34% of participants identifying as non-Hispanic white and the remaining identifying as another racial group or coded as multiracial. A majority (66%) identified as gay, about half (54%) were single, most (78%) were recruited from social media, and the sample had good geographic representation across all four major U.S. regions. By PrEP use status, 22% were currently taking PrEP, 17% had previously used PrEP, and 61% had never yet used PrEP (see Table 1).

**Table 1:** Demographics characteristics of young sexual minority men (n = 41).

Continuous Variables	*M*	*SD*
Age (range: 17–24)	21.0	2.5

Categorical Variables	*n*	%
Gender identity		
Cisgender man	35	85.4
Transgender man	6	14.6
Race/ethnicity		
Black, non-Hispanic	11	26.8
Latino or Hispanic	12	29.3
White, non-Hispanic	14	34.2
Multiracial/another	4	9.8
Sexual orientation		
Gay	27	65.9
Bisexual	12	29.3
Queer	2	4.9
Relationship status		
Single	22	53.7
Partnered	19	46.3
Pre-exposure prophylaxis (PrEP) use status		
Never	25	61.0
Prior PrEP use	7	17.1
Current PrEP use	9	22.0
Region		
Midwest	12	29.3
Northeast	11	26.8
South	12	29.3
West	6	14.6
Recruitment source		
Social media	32	78.1
Men-for-men geosocial networking apps	9	22.0

Percentages may not add up to 100 due to rounding.

Our thematic analysis identified five key themes related to LAI-PrEP decision-making: (1) concerns about adherence to LAI-PrEP dosing and clinic appointments, (2) awareness and knowledge of LAI-PrEP safety and efficacy data, (3) comfort with needles, (4) minimizing stigma associated with PrEP strategies more broadly, and (5) considerations of self-injection. Using constant comparison, we found experienced PrEP users approved of LAI-PrEP more than non-users. YSMM also found self-injection more acceptable if provided training, and self-injection was described as a convenient and empowering option to avoid PrEP stigma and discrimination.

As participants reflected upon the addition of LAI-PrEP to the currently available PrEP options, YSMM broadly acknowledged more PrEP options as beneficial to supporting uptake and persistence. A participant who previously used oral PrEP noted the importance of more options available to consumers, allowing more contextual fit to an individual’s life. Importantly, he noted LAI-PrEP and oral PrEP each offered different advantages and disadvantages for an individual to weigh.

*“I think [/HIV/] prevention is a lot like birth control. It's most helpful when there are a lot of options that fit different people's lives better. I don't think one is better; I think some are better for certain people.”* – 19-year-old, multiracial bisexual transgender man, prior PrEP use

### Concerns about adherence to LAI-PrEP dosing and clinic appointments

When participants weighed the advantages and disadvantages of LAI-PrEP compared to oral PrEP (both daily and event-driven PrEP), YSMM discussed the importance of adherence to PrEP dosing and clinic appointments, and how requirements of each strategy influenced their opinion. One participant who had never used PrEP noted concerns about committing to LAI-PrEP in the context of every 8-week dosing but felt that these concerns would be alleviated with using oral PrEP first before switching to LAI-PrEP. Importantly, this participant described the ideal—and tested—strategy to initiate LAI-PrEP, where oral cabotegravir is first used to identify any unanticipated side effects of the drug before providing a non-removable injection of the long-acting cabotegravir formulation.

Interviewer: *“What type of concerns do you think people will have about receiving PrEP by injection?”*

*“Commitment would be my main concern, though this could be solved by testing the PrEP pill first and then switching to the injection.”* – 19-year-old, Latino bisexual cisgender man, never used PrEP

Participants also noted the rigorous requirements of oral PrEP, where clinic visits are required quarterly for HIV and sexually transmitted infection testing. Moving to an every 8-week clinic visit was not considered to be more burdensome for some. However, another participant found the frequency of injections to be too much given their dislike of shots and would prefer injections twice a year at most. On a day-to-day basis, though, LAI-PrEP was found to require less upkeep, without the need for daily or event-driven pill adherence. When asked about receiving an injection every 8 weeks compared to a daily pill, participants expressed willingness to use LAI-PrEP if it was as effective as oral PrEP:

*“I mean I have to get bloodwork every three months anyway for [daily oral] PrEP... so yeah I would probably be interested in doing that method if it was proved to be equally effective in HIV prevention.”* – 23-year-old, White gay cisgender man, currently uses oral PrEP

*“I personally hate shots, but I can deal [/with/] it with them if the effectiveness period is a good length of time, at least 6 months or so. I could do 2 shots a year at most.”* – 21-year-old, Black gay cisgender man, never used PrEP

*“I would be interested in getting a shot as it would require less upkeep...”* – 19-year-old, White gay cisgender man, never used PrEP

### Awareness and knowledge of LAI-PrEP safety and efficacy data

Participants did not make decisions about LAI-PrEP in isolation. As reported by a participant above, decisions about oral and LAI-PrEP were considered collectively, such that he did not find the added clinic visits for LAI-PrEP to be burdensome if LAI-PrEP provided comparable protection to oral PrEP. Another participant described the importance of pill adherence for oral PrEP to be effective, and within the context of potentially missing a dose, he preferred an injection every 8 weeks. However, it is worth noting four or more oral PrEP doses per week provides adequate protection against HIV among cisgender men.

Interviewer: *“If someone only had to get a shot once every 8 or more weeks to receive PrEP, do you think people would prefer that method of PrEP over a pill? Why or why not?”*

*“Yes, because it's much easier, timewise. If you forget PrEP any day, the success rate goes down. I'd rather take a needle once every 8 weeks.”* – 22-year-old, Latino gay cisgender man, never used PrEP

In addition to information regarding efficacy, participants also wanted to know more about what was in LAI-PrEP injections and how the medicine stayed in the system for longer durations.

*“I think I’d feel better injecting myself, but I’d still fear what I’m injecting myself with.”* – 24-year-old, Black gay cisgender man, never used PrEP

*“I would prefer an injection, though I am curious as to how the medicine would remain in my system for 8 weeks when pills couldn’t.”* – 19-year-old, Latino bisexual cisgender man, never used PrEP

Nonetheless, LAI-PrEP efficacy was balanced in the context of the duration of efficacy, where another participant thought an injection-based PrEP was convenient, but he thought event-driven PrEP might be a better option, given his current frequency of sex, unless the protection lasted a year or greater. He didn’t feel the increased number of clinic visits was worthwhile for only two months of protection.

*“I love shots. They're so easy. I would do that if the effectiveness were boosted to a year*+*, otherwise, event-based is maybe better for me. My sex life is not consistent enough for the [/8/] weeks to ‘pay off’ every time.”* – 24-year-old, White queer cisgender man, prior oral PrEP use

### Comfort with needles

Participants also weighed the advantages and disadvantages between LAI-PrEP and oral PrEP in the context of their comfort with needles. Although reported relatively infrequently, discomfort with needles was a barrier to considering LAI-PrEP for some men. Participants reflected on their comfort with needles, but also the perceptions of others, including the context of concerns about blood exposure—relevant in the context of HIV prevention.

*“That would be ideal if I wasn’t so uncomfortable with needles.”* – 17-year-old, White bisexual transgender man, never used PrEP

*“I don’t think people would prefer the needle due to the fear with needles and fluid exposure.”* – 19-year-old, Latino bisexual cisgender man, never used PrEP

Although only described by one person, injections were considered synonymous to vaccines. He thought folks who did not believe in vaccines would also not believe in the protection afforded by an injectable version of PrEP. Instead, pills provided more reassurance to what individuals were putting in their bodies.

*“I think pills would be more reassuring to people, especially in the "anti-vax" group.”* – 18-year-old, White bisexual cisgender man, prior oral PrEP use

### Minimizing PrEP stigma

Participants discussed the continued pervasiveness of PrEP stigma broadly in their communities, despite their positive beliefs about the benefits of oral and LAI-PrEP. The choice in PrEP options was considered within the context of minimizing this PrEP stigma. As oral PrEP is frequently stereotyped as being for men who have sex with men, participants discussed the ability to avoid pills and pill bottles that could potentially out themselves to family or roommates. Many of these YSMM moved back home with their family during COVID-19, making this concern even more prominent within the current context of their lives. Moreover, participants also discussed the number of people they are required to be in contact with to remain on their oral PrEP regimen, which includes clinic visits and interacting with pharmacy employees. Thus, self-injection was even more appealing in the context of reducing PrEP stigma when considering LAI-PrEP.

*“I could see people who would, because maybe if you’re someone who’s trying to hide it, a pill bottle is a risk of being outed. Or maybe you don’t like taking pills every day.”* – 17-year-old, Latino gay transgender man, never used PrEP

### Considerations of self-injection

Participants discussed the possibility of self-administering LAI-PrEP, which was thought to limit the need for routine clinic visits and be more cost effective. Cost beyond the price of the medication was considered, as participants noted the potential expense of more frequent clinic visits. Realizing the potential impact of clinic-based fees, participants noted that self-injections administered at home would help avoid clinics and associated costs.

*“I dislike injections, but I think people would prefer that method in general. If you had to go into your doctor every eight weeks, it could potentially be expensive.”* – 23-year-old, White bisexual transgender man, never used PrEP

Participants expressed a range of opinions about self-injection. While some participants had major reservations about self-administered LAI-PrEP, many thought it could potentially be useful for others. Still others appreciated the option of self-injection, as some folks already use self-injections for other medications and found it potentially convenient if access to care is a challenge.

Interviewer: *“If you could inject yourself rather than going to a provider every 8 weeks, would that increase your willingness to use it?”*

*“I would not trust myself at all to inject myself honestly”* – 22-year-old, Latino gay cisgender man, never used PrEP

*“Yes, as I already do self-injections on a weekly basis”* – 23-year-old, White bisexual transgender man, never used PrEP

*“Eh. Self injections suck and are scary, but for certain people who have difficulty with accessing care, it would definitely be a bonus.”* – 19-year-old, multiracial bisexual transgender man, prior oral PrEP use

As described below, participants noted the importance of being provided training to self-administer the injection, with practical guidance including initial injection with a healthcare provider and the incorporation of written instructions with pictures or instructional videos.

*“Nope, I’ve never self injected, so I’m not afraid but would definitely need some very thorough instructions with pictures and videos to feel comfortable enough to get it done.”* – 21-year-old, Black gay cisgender man, never used PrEP

Moreover, another provided practical guidance about how self-injection could be made easier for YSMM, including designing the LAI-PrEP delivery device to be like those used to administer epinephrine for severe allergies (i.e., trade name: Epi-Pen), as quoted previously. This was particularly relevant within the context of the fear of needles and fluid exposure to make self-injection more accessible.

## Discussion

In this study, we sought to explore LAI-PrEP decision-making in the context of other available PrEP options among a nationwide sample of YSMM 17–24 years old, as well as the potential impact of a self-injection option. Preferences and decision-making about LAI-PrEP varied widely among YSMM, with participants weighing the advantages and disadvantages of each PrEP strategy within the context of their lives or others. Our thematic analysis identified decision-making to be centered around adherence, awareness and knowledge, comfort with needles, minimizing PrEP stigma, and considerations of self-administration, as YSMM weighed perceptions of the strengths and weaknesses of LAI-PrEP in comparison to the currently available oral PrEP regimens. Even when participants had initial negative reactions to LAI-PrEP, these negative perceptions were frequently considered in conjunction with its unique strengths, indicating acceptability of LAI-PrEP is very individualized and potentially amenable to decision aid intervention strategies. Importantly, we found LAI-PrEP to be appealing for all individuals, including specific subpopulations of YSMM with lower uptake of oral PrEP—such as Black, Latino, and transgender YSMM^49^—indicating the potential for LAI-PrEP to be considered among YSMM who are not currently taking oral PrEP.

Our findings indicate that LAI-PrEP could potentially reduce experiences of PrEP-related stigma, with implications for increasing overall PrEP uptake and persistence. It has been well-documented that the social context in which PrEP is used among YSMM is challenged by the negative perceptions attached to PrEP^50,51^, which can deter adoption, adherence, and persistence^52^. YSMM in our study discussed reservations about PrEP because of concerns about being presumed living with HIV or engaging in “risky” sexual behavior, consistent with prior research^51–54^. As such, the administration of LAI-PrEP in the privacy of a clinical setting and eliminating the need for prescription bottles after initiation could be a significant motivator among some YSMM. Especially relevant to the ongoing COVID-19 pandemic, LAI-PrEP could assuage privacy concerns for people who live at home with their families who worry about being outed by finding pills^42^. However, more research is needed to better understand how LAI-PrEP could reduce experiences of PrEP-related stigma and to understand the stigma-related perceptions of people who will choose LAI-PrEP over oral based PrEP once it is available to consumers.

LAI-PrEP has the potential to increase PrEP access for YSMM with concerns about adherence to oral PrEP, including daily and event-driven options. However, accessing LAI-PrEP would still be an intensive process, with bi-monthly clinic visits for injections in addition to follow-up laboratory work. Therefore, self-administered LAI-PrEP could be beneficial in providing greater utility for this method of PrEP delivery. Participants in this study expressed mixed opinions related to self-administration. Some felt it would increase ease of access and use as well as safeguard privacy compared to oral PrEP, while others described their fear of incorrectly administering the injection or fear of needles in general as barriers to self-administration. Instructions and other training materials could be provided with LAI-PrEP for self-administration, which has been a successful approach in HIV self-testing^55,56^. Providing the option of self-administered LAI-PrEP could potentially increase PrEP access and decrease the burden on patients for adherence, and cited barriers could be overcome with training to increase comfort with self-administered LAI-PrEP.

The dosing schedule of cabotegravir for LAI-PrEP and necessary follow-up visits were described as potential barriers by prescribing providers^57^, which align with our patient-level results indicating the first available LAI-PrEP using cabotegravir may not provide a duration of protection long enough to warrant consideration over oral PrEP strategies. Participants requesting once- or twice-yearly injections as more desirable to an every 8-week schedule. Additionally, socioeconomic barriers may play a large role in access and persistence. Insurance coverage, or lack thereof, may hinder LAI-PrEP acquisition, as prescription requires an initial clinical visit with follow-up and lab visits that may or may not be covered based on the planning resources of clinics^58,59^. Further, LAI-PrEP could increase financial burden for individuals as insurance companies may choose to cover generic oral PrEP over LAI-PrEP, especially in the context of current cost-effectiveness modeling that indicates cabotegravir could be prohibitively expensive based on the current price of cabotegravir combinations used for HIV treatment^60^.

Findings from this study contribute to the growing literature about LAI-PrEP, with implications for future implementation of LAI-PrEP. Researchers have identified the potential benefit of adding LAI-PrEP to the available PrEP options, as many current PrEP users prefer LAI-PrEP over oral PrEP regimens^20–22^. Based on these reports, it is estimated that up to two-thirds (66.7%) of oral based PrEP users may consider switching to LAI-PrEP when it becomes available^20–22^. Prior research also demonstrated the potential role of LAI-PrEP in increasing PrEP uptake overall, as many individuals who could benefit from some form of PrEP preferred LAI-PrEP to oral PrEP^23–30^. In fact, preliminary evidence indicates LAI-PrEP may be more preferrable for several marginalized, priority populations left behind in oral PrEP uptake, such as young Black SMM^28^, Black women^29^, and people who inject drugs^30^. Our research adds a more nuanced interpretation about the potential appeal of LAI-PrEP. The consideration of LAI-PrEP was rarely without some reservations or need for additional information, suggesting prior survey research may over- or under-estimate the potential uptake of LAI-PrEP.

Shared decision-making between patients and their providers is needed^39^, but both need to be adequately informed to help make those decisions. We found YSMM had questions about the safety and efficacy of LAI-PrEP compared to oral PrEP, as well as concerns about cost. This is particularly relevant in the context of newly available generic oral PrEP and its Grade A recommendation by the U.S. Preventive Services Task Force^61^, which requires insurance companies to fully cover the cost of PrEP medication and applicable clinical and laboratory costs. LAI-PrEP implementation may be delayed unless covered by these same policies. Moreover, YSMM would also benefit from further discussion about how LAI-PrEP is initiated and discontinued, as concerns about adherence to dosing and follow-up appointments influenced their decision-making. Unfortunately, prior research shows a continued need for provider training in the realm of HIV prevention, as lack of knowledge is a significant barrier to PrEP implementation among providers of all kinds^62^.

Our research is not without limitation. First, recruitment occurred online, which could limit generalizability to YSMM without internet access. However, the online focus group discussion platform was also accessible by smartphones, reducing some concerns about access, and no participants had connectivity issues that could not be resolved with the research team. Second, synchronous online focus group discussions were conducted via an online chat platform, which did not allow the research team to use non-verbal cues to prompt probing to facilitate discussion. Nonetheless, we do not believe this affected the quality of our data or findings^45,46^. Lastly, our sample comprised of mostly cisgender gay and bisexual men, potentially limiting generalizability; however, we found no differences by gender (cisgender/transgender) or sexual identity (gay/bisexual/queer) within the sample during constant comparison analyses.

## Conclusions

In summary, we found preferences and decision-making about LAI-PrEP varied widely among YSMM, with participants weighing the advantages and disadvantages of LAI-PrEP within the context of their lives. Decision-making centered around themes about adherence, awareness and knowledge, comfort with needles, minimizing PrEP stigma, and considerations of self-injection, which provides data to support implementation activities when intramuscular cabotegravir becomes available. Future research should work to develop decision-making tools to support implementation and guide patient choice and shared decision-making with providers.

## Data Availability

Data is available upon request to the Administrative Core of the Center for AIDS Intervention Research. Individuals who meet criteria for access to de-identified data should contact the Principal Investigator (sjohn@mcw.edu) or Kevin Brown (kdbrown@mcw.edu) to facilitate data transfer.
